# Deep exploration and precise identification of key risk factors for diabetic peripheral neuropathy using the random forest algorithm

**DOI:** 10.3389/fendo.2025.1740545

**Published:** 2026-01-07

**Authors:** Yongnan Li, Yongsheng Li, Gan Sen, Bin Hu

**Affiliations:** 1 Department of Nursing, Suzhou BenQ Medical Center, Suzhou, China; 2 Department of Preventive Medicine, Medical College, Tarim University, Alar, China; 3 College of Medical Engineering and Technology, Xinjiang Medical University, Urumqi, China; 4 Institute of Medical Engineering Interdisciplinary Research, Xinjiang Medical University, Urumqi, China

**Keywords:** 25(OH)D3, diabetic peripheral neuropathy, GPS, random forest, type 2diabetes mellitus (T2DM)

## Abstract

**Background:**

Diabetic peripheral neuropathy (DPN) is a prevalent and highly disabling complication of diabetes mellitus, associated with markedly increased rates of disability and mortality. Timely intervention and effective management have been consistently shown to substantially reduce the risk of DPN onset and progression.

**Methods:**

This retrospective cohort study analyzed 1, 004 hospitalized patients with type 2 diabetes mellitus (T2DM) admitted to the endocrinology department of a hospital in Jiangsu Province, China. A risk prediction model for DPN was developed using the Random Forest (RF) algorithm, while logistic regression analysis was employed to identify the major risk factors. The overarching aim was to provide a reliable risk assessment tool for clinical application.

**Findings:**

Five principal factors were identified as significantly associated with DPN risk: age (OR = 1.257, 95% CI [1.188–1.367], p < 0.001), serum 25(OH)D3 levels (OR = 0.791, 95% CI [0.759–0.854], p < 0.001), duration of diabetes (OR = 1.431, 95% CI [1.285–1.617], p < 0.001), glycated hemoglobin (HbA1c) (OR = 1.236, 95% CI [1.197–1.391], p < 0.001), and glycated serum protein (GSP) (OR = 1.091, 95% CI [1.047–1.201], p = 0.017). A DPN risk prediction model incorporating these variables achieved an area under the receiver operating characteristic curve (AUC) of 0.829 (95% CI: 0.802–0.857), demonstrating excellent discriminatory performance.

**Interpretation:**

The Random Forest–based DPN risk prediction model successfully identified five critical risk factors, offering a solid theoretical foundation for personalized strategies in DPN prevention and management among patients with diabetes. This model exhibits high predictive utility in clinical practice.

## Introduction

Diabetes has become one of the most serious and common chronic diseases in today’s society. It not only leads to life-threatening complications with a high rate of disability but also incurs significant treatment and management costs, significantly shortening patients’ life expectancy (1). According to statistics from the International Diabetes Federation (IDF), the global prevalence of diabetes has reached epidemic levels. In 2021, approximately 537 million adults (aged 20-79) were living with diabetes, accounting for about 10% of the global population. It is estimated that by 2030, the number of affected individuals will increase to 643 million, and by 2045, this number will further rise to 783 million. During this period, despite an expected 20% growth in the global population, the number of people with diabetes will increase by 46% (2, 3), creating a significant disease burden on society.

Diabetic Peripheral Neuropathy (DPN) is a common and severely detrimental complication of diabetes, with an incidence rate as high as 50% (4). In its early stages, DPN often presents with no or mild non-specific symptoms, which are usually only detectable through specific tests (5, 6). If not identified and managed early, severe complications such as foot ulcers and gangrene may develop, ultimately leading to an increased risk of amputation (7). Therefore, early diagnosis and intensive intervention are crucial for improving subclinical DPN and reducing the risk of its progression to clinical DPN. However, early diagnosis remains a major challenge in clinical practice.

Currently, most studies focused on the assessment and prediction of DPN primarily concentrate on symptoms and signs that have already manifested in patients, such as limb numbness, pain, and foot ulcers. However, recent research indicates that DPN may also occur in patients with prediabetes. In these individuals, up to 50% exhibit no obvious symptoms, which leads to a failure to identify the condition in a timely manner (8, 9). In recent years, machine learning methods have demonstrated significant value in the early identification and risk prediction of DPN (10, 11). These approaches can extract underlying patterns from multidimensional clinical data, thereby enhancing the accuracy and efficiency of predictions. Among various machine learning algorithms, the Random Forest (RF) model stands out as an effective method for handling nonlinear relationships and missing data. It assigns importance scores to each feature variable, enabling the identification of variables that exert a critical influence on classification outcomes (12, 13). Moreover, the RF method does not require the consideration of multicollinearity or complex variable selection processes.

This study aims to apply the RF method in conjunction with logistic regression to identify the primary risk factors for DPN in diabetic patients and to develop a practical DPN risk prediction model. The goal is to provide clinical practitioners with a powerful decision-support tool.

## Methods

### Study design and population

This study is a retrospective observational study that collects data from the Electronic Medical Records (EMR) and digital medical record systems. A total of 1, 004 patients with Type 2 Diabetes Mellitus were included in the study, all of whom were hospitalized in the endocrinology department of a hospital in Jiangsu Province from July 2021 to December 2023. The study protocol was approved by the Ethics Committee of Suzhou BenQ Hospital (SZMJYY2022102001) and informed consent was waived. The research was carried out in accordance with relevant guidelines and regulations.

Inclusion criteria were as follows: (a). patients between 20–79 years old, (b). the diagnostic criteria for T2DM was based on the Guideline for the prevention and treatment of type 2 diabetes mellitus in China (2020 edition) (14), or (c). all research participants were able to communicate independently. Exclusion criteria were as follows: (a). incomplete clinical data, and (b). informed consent was unsigned.

Data completeness was high; variables with missing values (less than 5% for any single variable) were imputed using the median for continuous variables and the mode for categorical variables to ensure robustness of the model.

### Data collection

All baseline clinical characteristics, including age, leukocyte, neutrophil, eosinophil, lymphocyte, hemoglobin, platelet, total cholesterol, high-density lipoprotein, low-density lipoprotein, direct bilirubin, unconjugated bilirubin, total bilirubin, aspartate aminotransferase, alanine aminotransferase, body mass index, systolic blood pressure, diastolic blood pressure, serum creatinine, hemoglobin A1c, glycosylated serum protein, apolipoprotein A1, apolipoprotein B, fasting blood glucose, triglyceride, blood urea nitrogen, cystatin C, 1-hour postprandial blood glucose, 2-hour postprandial blood glucose, 25-hydroxyvitamin D3, blood glucose, homocysteine, alcohol, gender, nation, smoking, diabetic peripheral neuropathy [The diagnostic criteria for diabetic peripheral neuropathy was based on the Guideline for the prevention and treatment of type 2 diabetes mellitus in China (2020 edition)].

Diagnostic criteria included: (a) abnormal temperature perception (tested with a Tip-therm), (b) abnormal vibration perception (tested with a 128-Hz tuning fork), (c) decreased or absent sensation on nylon filament examination (10-g monofilament), (d) absent ankle reflex, and (e) abnormal nerve conduction velocity (NCV). NCS was performed by trained technicians using standard electromyography equipment. Abnormal NCV was defined as values below the lower limit of the laboratory’s reference range (adjusted for age and height). A diagnosis of DPN required the presence of at least two abnormal findings from criteria (a)-(d) OR one abnormal clinical sign plus abnormal NCV (criterion e). All assessments were performed during hospitalization, and data were extracted from structured electronic medical records.

### Statistical analysis

For continuous variables that follow a normal distribution with homogeneity of variance, the results are presented as mean ± standard deviation (Mean ± SD), and comparisons are made using independent sample t-tests. If the data do not follow a normal distribution, the results are described using the median and interquartile range (P25, P75), and comparisons are made using the rank-sum test. For categorical variables, the results are presented as frequency percentages (%) and comparisons are made using chi-square tests or Fisher’s exact test. The Random Forest (RF) method combined with logistic regression analysis is used to screen for significant predictive variables. All statistical tests are two-tailed, with a significance level set at 0.05. All statistical analyses are performed using R software (version 4.2.1; accessed on October 31, 2022, at https://www.r-project.org).

Random Forest: Random Forest (RF) is an ensemble learning method based on decision tree classifiers, widely used in the field of bioinformatics. The basic principle is as follows: if the original training set contains N samples with M features, RF selects N samples from the original training set using Bootstrap resampling and randomly selects M features to train a fully grown decision tree. This process is repeated multiple times to generate a set of decision trees. Ultimately, RF aggregates the outputs of these decision trees into an ensemble model and generates the final prediction through a voting mechanism. Therefore, the number of decision trees and the randomly selected features are crucial for building an accurate RF model. RF model was implemented using the `randomForest` package in R. Key hyperparameters were optimized via out-of-bag (OOB) error estimation and 10-fold cross-validation. The final model used 500 trees (`ntree=500`), and the number of variables randomly sampled as candidates at each split (`mtry`) was set to 5 (approximately the square root of the total number of predictors). The Gini impurity index was used for node splitting. Model performance was evaluated using the OOB error estimate and the area under the ROC curve (AUC) derived from the cross-validation procedure.

## Results

### Patient characteristics

A total of 1, 004 Type 2 Diabetes Mellitus (T2DM) patients were included in this study. **Table 1** presents the detailed baseline demographic and clinical characteristics of the two groups. Among them, 515 patients were diagnosed with Diabetic Peripheral Neuropathy (DPN), with an incidence rate of 51.29%. Of the DPN patients, 305 were male (59.22%) and 210 were female (40.78%) (**Table 1**).

**Table 1:** Baseline characteristics of all patients.

Variables	No DPN (N = 489)	DPN(N = 515)	p
AGE	52.00 (46.00, 60.00)	60.00 (53.00, 68.00)	<0.001
Leukocyte	6.78 (5.65, 8.38)	6.50 (5.33, 7.78)	<0.001
Neutrophil	3.81 (3.03, 4.95)	3.70 (2.89, 4.65)	0.048
Eosinophil	0.13 (0.08, 0.22)	0.13 (0.08, 0.21)	0.834
Lymphocyte	2.17 (1.72, 2.66)	1.98 (1.56, 2.46)	<0.001
Hemoglobin	142.00 (128.00, 153.00)	137.00 (126.00, 148.50)	<0.001
Platelet	221.00 (181.00, 259.00)	216.00 (181.50, 257.50)	0.62
TC	4.13 (3.58, 4.85)	4.14 (3.38, 4.79)	0.163
HDL	1.02 (0.84, 1.21)	1.09 (0.90, 1.29)	<0.001
LDL	2.61 (2.17, 3.30)	2.66 (2.03, 3.21)	0.176
DB	3.30 (2.20, 4.70)	3.22 (2.34, 4.50)	0.706
UB	7.50 (5.30, 10.18)	6.61 (4.58, 9.98)	0.014
TB	11.00 (8.20, 14.09)	10.00 (7.60, 13.95)	0.029
AST	18.20 (14.80, 24.50)	17.30 (14.10, 21.41)	<0.001
ALT	22.44 (15.30, 32.41)	18.67 (13.90, 26.64)	<0.001
BMI	25.72 (23.77, 28.41)	25.39 (23.23, 27.93)	0.022
SBP	122.00 (115.00, 135.00)	128.00 (118.00, 140.00)	0.008
DBP	80.00 (70.00, 82.00)	78.00 (70.00, 80.00)	0.147
Duration of T2DM	4.00 (2.00, 10.00)	12.00 (6.00, 17.00)	<0.001
Scr	67.00 (55.00, 80.00)	66.00 (54.00, 80.00)	0.350
HbA1C	7.80 (6.70, 9.50)	9.10 (7.60, 10.90)	<0.001
GSP	2.53 (2.23, 3.03)	2.77 (2.41, 3.29)	<0.001
ApoA1	1.13 (0.99, 1.29)	1.16 (1.02, 1.33)	0.021
ApoB	0.92 (0.75, 1.10)	0.88 (0.74, 1.08)	0.118
FBG	7.71 (6.42, 9.92)	8.91 (6.96, 10.79)	<0.001
TG	1.85 (1.21, 2.85)	1.60 (1.10, 2.50)	0.002
BUN	5.20 (4.30, 6.50)	5.40 (4.40, 6.50)	0.445
Cys C	0.71 ± 0.21	1.45 ± 0.37	<0.001
Obs	14.87 (12.81, 17.17)	15.22 (13.18, 17.67)	0.091
PBG	17.39 ± 4.51	18.85 ± 4.48	<0.001
25(OH)D3	15.32 ± 5.48	11.76 ± 5.16	<0.001
BG	7.65 (6.12, 10.57)	9.40 (6.50, 13.29)	<0.001
Hcy	11.09 ± 2.77	15.71 ± 3.21	<0.001
Alcohol			0.178
No	145(29.65%)	132(25.63%)	
Yes	344(70.35%)	383(74.36%)	
Gender			0.021
Male	324 (66.26%)	305 (59.22%)	
Female	165 (33.74%)	210 (40.78%)	
Nation			0.269
Ethnic Han	425 (86.91%)	435(84.47%)	
Other	64 (13.09%)	80(15.53%)	
Smoking			0.090
No	205 (41.92%)	189 (36.70%)	
Yes	284 (58.08%)	326 (63.30%)	
Upn			0.672
–	437 (89.37%)	448 (86.99%)	
+	17 (3.48%)	20 (3.88%)	
++	16 (3.27%)	20 (3.88%)	
+++	19 (3.89%)	27 (5.24%)	

DPN, Diabetic peripheral neuropathy; M, Male; F, Female; TC, Total cholesterol; HDL, High-density lipoprotein; LDL, Low-density lipoprotein; DB, direct bilirubin; UB, unconjugated bilirubin; TB, total bilirubin; AST, aspartate aminotransferase; ALT, alanine aminotransferase; BMI, Body mass index; SBP, Systolic blood pressure; DBP, Diastolic blood pressure; Scr, Serum creatinine; HbA1c, hemoglobin A1c; GSP, glycated serum protein; ApoA1, apolipoprotein A1; ApoB, apolipoprotein B; FBG, fasting blood glucose; TG, Triglyceride; BUN, Blood urea nitrogen; Cys C, Cystatin C; Obs, 1-hour postprandial blood glucose; PBG, 2-hour postprandial blood glucose; 25(OH)D3, 25-hydroxyvitamin D3; BG, blood glucose; Hcy, homocysteine; Upn, urinary protein.

### Characteristics’ selection

**Figure 1:**
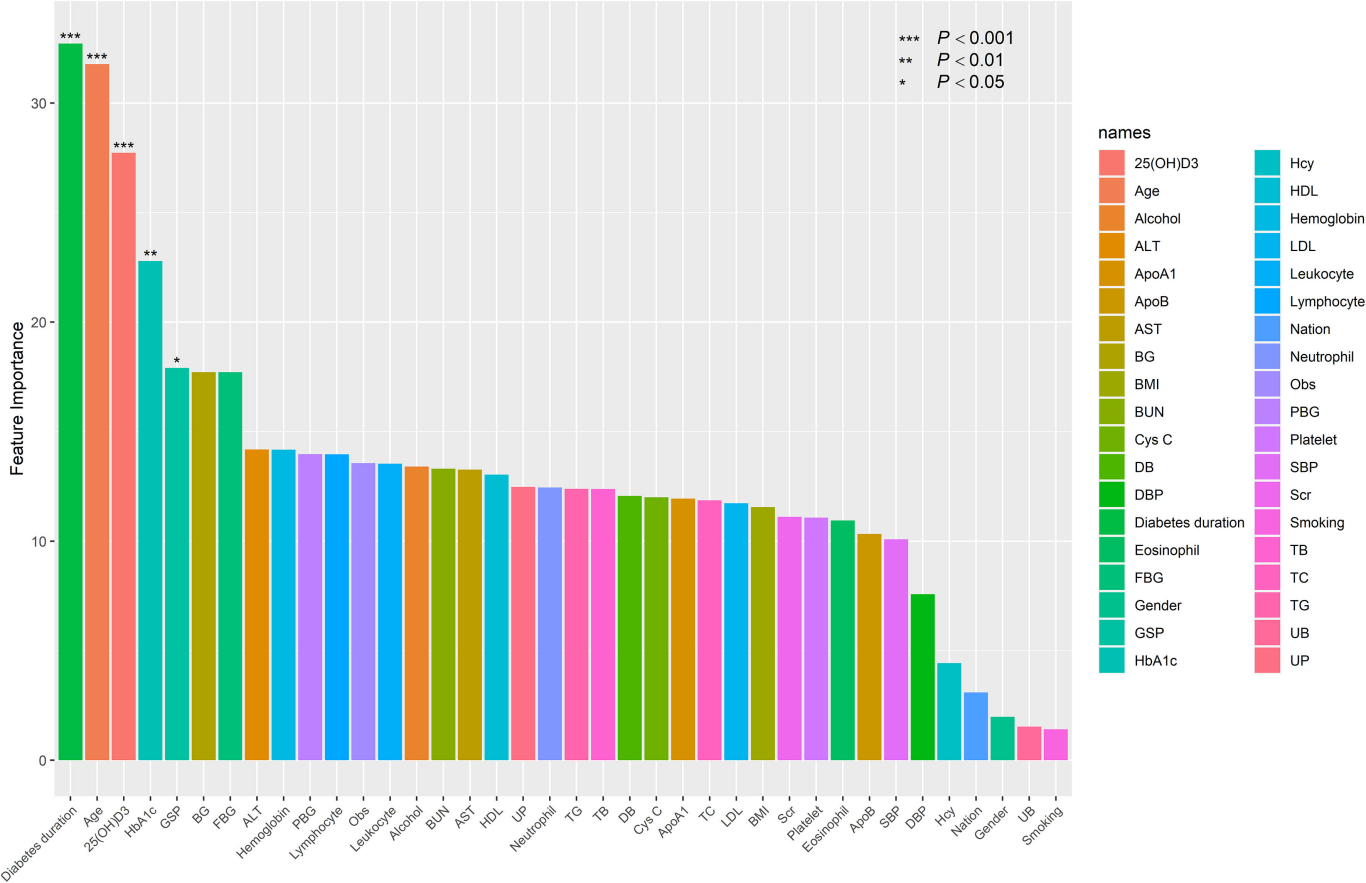
Feature importance ranking in RF.

During the construction of the RF model, we optimized key parameters (setting the number of random features to 5 and the number of decision trees to 500) and trained the model through repeated resampling of all samples, thereby generating the corresponding RF model. Subsequently, the importance scores of the model variables were ranked. The higher the score, the greater the impact of the corresponding variable on model classification. Through this process, we identified five significant variables (**Figure 1**).

These potential predictive factors included age, 25(OH)D3, duration of diabetes, HbA1c, and GSP. We then performed multivariate logistic regression analysis on these five candidate variables. Ultimately, age (OR = 1.257, 95% CI [1.188, 1.367], p < 0.001); 25(OH)D3 (OR = 0.791, 95% CI [0.759, 0.854], p < 0.001); duration of diabetes (OR = 1.431, 95% CI [1.285, 1.617], p < 0.001); HbA1c (OR = 1.236, 95% CI [1.197, 1.391]); and GSP (OR = 1.091, 95% CI [1.047, 1.201], p = 0.017) were all statistically significant and were thus selected for the development of the predictive model (**Table 2**). There was no multiple collinearity among the risk factors included in the model, and the maximum variance expansion factor (VIF) was 1.766 while the lowest was 1.099. Therefore, we construct the clinical risk prediction model with these 5 statistically significant variables.

**Table 2:** Multivariate logistic regression analysis for risk factors of DPN.

Variables	OR	95% CI	Pr(>|z|)
AGE	1.257	(1.188, 1.367)	< 0.001
Duration of T2DM	1.431	(1.285, 1.617)	< 0.001
25(OH)D3	0.791	(0.759, 0.854)	< 0.001
HbA1C	1.236	(1.197, 1.391)	< 0.001
GSP	1.091	(1.047, 1.201)	0.017

25(OH)D3, 25-hydroxyvitamin D3; HDL, high-density lipoprotein; HbA1c, hemoglobin A1c; GSP, glycated serum protein.

**Figure 2:**
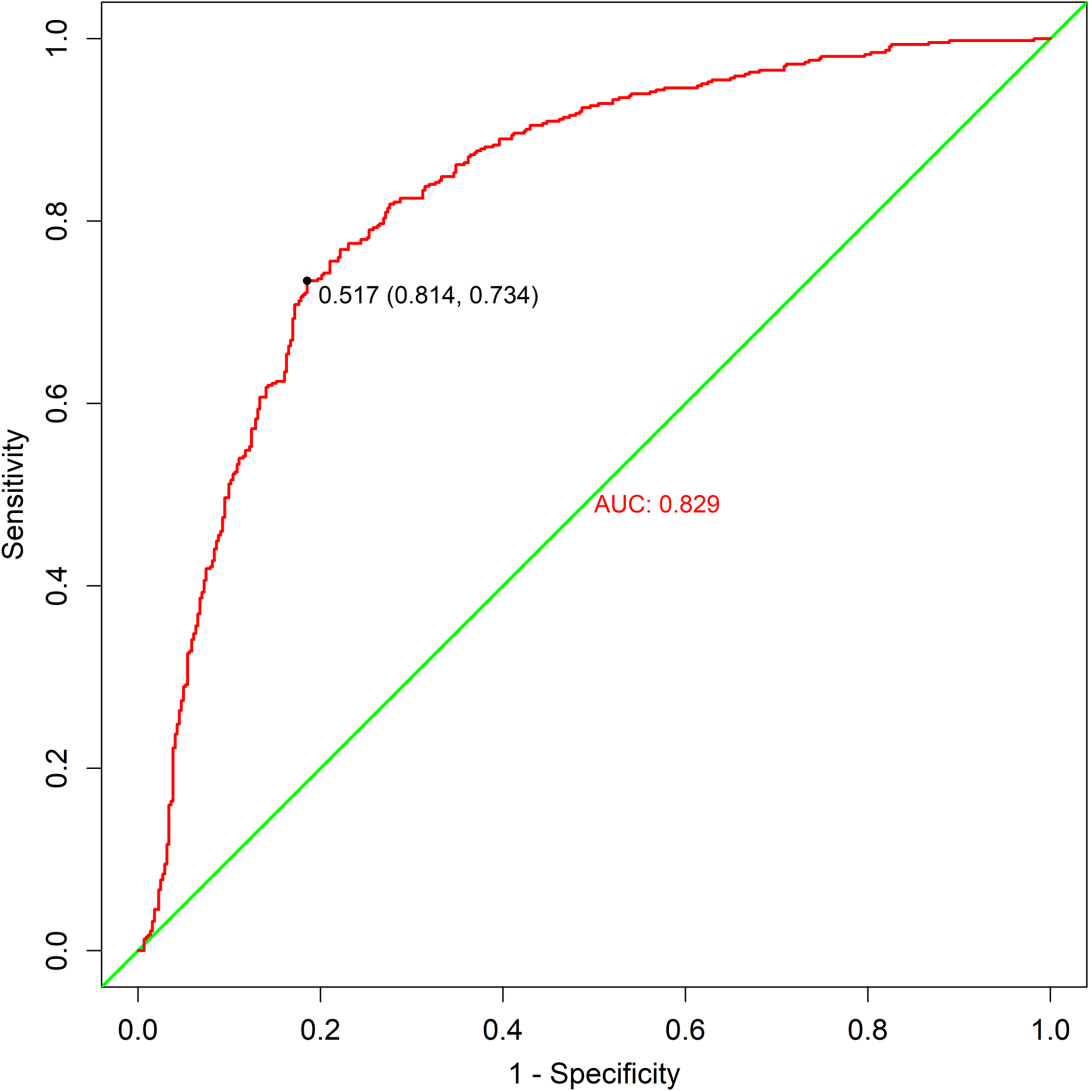
ROC curves. ROC, receiver operating characteristic.

In this study, the five selected variables demonstrated significant predictive accuracy, with an AUC value of 0.829 (95% CI: 0.802-0.857), sensitivity of 0.734, and specificity of 0.814 (as shown in **Figure 2**).

## Discussion

In this study, the prevalence of Diabetic Peripheral Neuropathy (DPN) among all Type 2 Diabetes Mellitus (T2DM) patients was approximately 51.3% (515/1004). Analysis using the Random Forest (RF) model revealed that disease duration, 25(OH)D3 levels, age, HbA1c, and Glycated Serum Protein (GSP) were significant risk factors for the occurrence of DPN. The results of logistic regression analysis also indicated that these five factors were significant risk factors for DPN. Based on the identification of DPN risk factors by the RF model, its prediction accuracy of 82.9% suggests that this model holds high clinical value.

In most epidemiological studies on Diabetic Peripheral Neuropathy (DPN), the duration of diabetes and patient age, due to their immutable nature, have become key factors in the occurrence of DPN (15–17). As the duration of diabetes extends, patients’ age tends to increase as well, which further raises the likelihood of developing DPN. Notably, a specific cross-sectional study highlighted a significant positive correlation between the duration of diabetes and the prevalence of DPN, indicating that the onset of DPN is closely related to the duration of diabetes, typically occurring between 8 and 16 years of disease progression (16). Furthermore, a retrospective study also found that the duration of diabetes and patient age were the two most critical risk factors for DPN, particularly the duration of diabetes (17). Longer diabetes duration is often closely associated with chronic hyperglycemia, which activates various biochemical pathways, inducing oxidative stress, leading to diabetic neuronal damage and ischemia (18). One possible explanation is that patients may have already developed diabetes and DPN but, for various reasons, were not diagnosed in time. As the disease progresses, prolonged poor blood glucose control accelerates the onset of DPN (19). This finding helps explain why the duration of diabetes and age were selected as key predictive variables in the final model of this study.

In recent years, the relationship between vitamin D (primarily present in the body as 25(OH)D3) and microvascular complications of type 2 diabetes has garnered widespread attention from scholars both domestically and internationally. Among these complications, diabetic peripheral neuropathy (DPN) has emerged as a major area of research. Existing studies suggest a growing clarity in the correlation between vitamin D and DPN. One six-month prospective randomized controlled trial (20) included 150 patients with type 2 diabetes, with 50 patients receiving oral hypoglycemic treatment alone, 50 receiving empagliflozin treatment, and 50 receiving a combination of empagliflozin and vitamin D. The results demonstrated that the addition of vitamin D to empagliflozin treatment significantly improved the symptoms of diabetic neuropathy. Another prospective placebo-controlled trial (21) conducted by D. Shehab included a treatment group (n = 57) and a placebo group (n = 55), showing that short-term oral vitamin D3 supplementation effectively improved vitamin D levels and neuropathy symptoms in patients with type 2 diabetes. Additionally, several studies (22–24) consistently indicate a close relationship between DPN and vitamin D deficiency. In this study, serum 25(OH)D levels were significantly reduced in patients with type 2 diabetes and concurrent DPN, aligning with previous findings that vitamin D deficiency is associated with the onset of DPN.

Vitamin D, as a crucial neurotrophic factor, plays a vital role in the health of the nervous system. One of its actions is to upregulate the expression of the vitamin D receptor (VDR), thereby promoting the production of nerve growth factor (NGF). NGF is an essential protein for the development, maintenance, and repair of neurons in the peripheral nervous system (25, 26). Further research has shown that vitamin D deficiency may exacerbate diabetic peripheral neuropathy-related pain perception by increasing the number of axons containing calcitonin gene-related peptide (CGRP), which plays a critical role in pain conduction and regulation. In diabetic patients, this mechanism is closely linked to the manifestation of neuropathic pain. Thus, vitamin D deficiency may aggravate pain induced by nerve damage through the aforementioned pathways (26). Vitamin D, as a neurotrophic factor, promotes nerve health by upregulating neurotrophic factors such as nerve growth factor (NGF). Furthermore, vitamin D influences insulin sensitivity and secretion, and its deficiency is associated with insulin resistance, which may exacerbate metabolic dysfunction in diabetes. While vitamin D supplementation has shown promise in improving neuropathic symptoms in some studies, the causal relationship and optimal dosing require further investigation (27, 28). It is also noteworthy that there is a significant association between vitamin D deficiency and insulin resistance. Research (29) indicates that patients with vitamin D deficiency have a markedly increased risk of developing insulin resistance, which not only makes diabetes control more difficult but may also accelerate the progression of metabolic syndrome. It is crucial to note that the majority of evidence linking vitamin D to DPN remains observational and associative. Interventional trials have yielded mixed results, and publication bias may favor positive findings. Therefore, while our study identifies low 25(OH)D3 as a significant risk marker, it does not establish causality. The potential therapeutic role of vitamin D supplementation in preventing or treating DPN requires validation through large-scale, well-designed randomized controlled trials.

Currently, the pathogenesis of diabetic peripheral neuropathy (DPN) remains incompletely understood. The main hypotheses involve inflammation, oxidative stress, and mitochondrial dysfunction (30, 31). In type 2 diabetes mellitus (T2DM), key triggers for these metabolic events are hyperglycemia, and effective blood glucose control is fundamental to the successful management of T2DM and its complications (32). Existing research (33) highlights that one of the main features of T2DM patients is chronic hyperglycemia, and plasma glucose regulation is closely associated with the onset of DPN. Studies show a significant correlation between glucose variability and the occurrence of DPN in T2DM patients (34). Furthermore, elevated HbA1c levels (which reflect poor blood glucose control) have been confirmed to be significantly associated with an increased risk of DPN in diabetic patients (35). In fact, fasting blood glucose is an immediate diagnostic marker that can be influenced by various factors, while HbA1c, representing blood glucose control over the past 2–3 months, is considered a more reliable biomarker for blood glucose control (36). It is widely believed that the higher the HbA1c level, the greater the risk of DPN. The results of this study also show that an increase in HbA1c significantly raises the risk of DPN. However, HbA1c levels can be influenced by several factors, including anemia, hemoglobin lifespan, age, pregnancy, and racial differences. Additionally, because HbA1c reflects a “delayed effect” of blood glucose changes, it cannot capture recent fluctuations in blood glucose, whereas diabetic microvessels are more sensitive to short-term changes in blood glucose (37). In this context, glycated serum proteins (GSP) serve as a supplementary indicator and hold significant value. Due to the relatively short half-life of albumin (17–19 days), GSP can more accurately reflect a patient’s blood glucose control over the past 2–3 weeks. The results of this study indicate that GSP is an independent risk factor for DPN. Although we observed that for each 1-unit increase in GSP, the odds ratio (OR) for DPN risk was 1.091, its predictive ability for DPN incidence on its own may be limited. Previous studies have not identified GSP as an independent risk factor for DPN, likely because in those studies, HbA1c had a higher predictive value. Therefore, the potential mechanisms and predictive value of GSP in DPN occurrence still require further investigation.

The random forest (RF) model, composed of numerous decision trees, can automatically identify the most critical input variables and handle both continuous and categorical variables, making it widely used in clinical applications (38).

The existing research on prediction model of diabetes peripheral neuropathy (DPN) has included as many as nine predictive factors, including diabetes retinopathy and diabetes nephropathy (7). As we all know, these microvascular complications of diabetes often occur simultaneously with DPN, which has a strong correlation and significant impact. Existing research suggests that these microvascular complications share some common risk factors, such as HbA1c, age, and disease duration. If these comorbidities are included in the prediction model, it may lead to bias in the model’s predictions. Therefore, excluding the influence of these factors can more accurately reveal the pathogenesis of DPN and improve the reliability of its prediction model. In this study, we analyzed various risk factors and indicators associated with early DPN. By using the RF model to automatically identify relevant variables, we avoided the limitations of solely observing individual indicators, as individual markers may have biases and one-sidedness. Finally, combining multivariate logistic regression, we identified five risk factors, achieving an 82.9% predictive accuracy, which offers greater generalizability and practicality compared to previous studies (7, 17).

However, this study also has certain limitations. First, the retrospective, single-center design introduces risks of selection and information bias. Hospitalized patients may not be representative of the general T2DM population, potentially overestimating DPN prevalence and risk factor associations. Second, To some extent, the potential bias introduced by other microvascular complications, such as diabetic retinopathy and diabetic nephropathy, has not been ruled out. Therefore, in future studies, we will consider the impact of these comorbidities to more accurately reflect the progression of the disease in the real world. Thirdly, our study cohort consisted of hospitalized patients, who may represent a more severe spectrum of T2DM with potentially higher complication rates. The cross-sectional design limits causal inference, and pre-existing diagnoses or treatments could have influenced both risk factor levels and DPN status. To address these, our future research plan includes a prospective, multicenter cohort study to externally validate the model’s performance and generalizability across diverse healthcare settings.

To summarize, this study successfully identified five key risk factors for diabetic peripheral neuropathy (DPN) using the Random Forest (RF) model, providing a valuable basis for the early diagnosis and personalized management of DPN. The findings hold significant clinical implications.

## Data Availability

The data analyzed in this study is subject to the following licenses/restrictions: The data underlying this article will be shared on reasonable request to the corresponding authors. Requests to access these datasets should be directed to sengan99@163.com.
